# A Study of the Epidemiology, Clinical, and Phenotypic Characteristics of Inflammatory Bowel Disease in the Northen-Central Region of Saudi Arabia

**DOI:** 10.3390/diagnostics13132135

**Published:** 2023-06-21

**Authors:** Ahmed A. Al-Fawzan, Sulaiman A. Al-Radhi, Ahmed S. Al-Omar, Nawaf H. Al-Mutiri, Ammar M. Al-Ammari, Mohammad El-Gohary, Amal N. Shamsan, Hamdan M. Al Shehri, Naif S. ALGhasab

**Affiliations:** 1 Department of Internal Medicine, King Fahad Specialist Hospital, Burydah 52366, Saudi Arabia; 2 Gastroenterology and Endoscopy Unit, King Fahad Specialist Hospital, Burydah 52366, Saudi Arabia; 3 Department of Internal Medicine, Buraydah Central Hospital, Burydah 52361, Saudi Arabia; 4 Department of Internal Medicine, King Saud Hospital, Onaizah 56437, Saudi Arabia; 5 Department of Internal Medicine, Medical College, Najran University, Najran 61441, Saudi Arabia; 6 Department of Internal Medicine, Medical College, Ha’il University, Ha’il 55476, Saudi Arabia

**Keywords:** inflammatory bowel disease (IBD), ulcerative colitis (UC), Crohn’s disease (CD)

## Abstract

Background: Inflammatory bowel disease (IBD), including Crohn’s disease (CD) and ulcerative colitis (UC), is becoming increasingly prevalent in Saudi Arabia. However, there is limited data on the characteristics and manifestations of IBD in this population. This study aimed to establish a multi-center database of patients with IBD in the Qassim region of Saudi Arabia to better understand the demographics, prevalence, and manifestations of IBD in this population. Methods: This retrospective study included patients diagnosed with IBD at three healthcare facilities in the Qassim region of Saudi Arabia. The patient’s demographic and clinical characteristics, disease location and behavior, age at diagnosis, medication use, related surgeries, and extraintestinal manifestations were recorded based on the Montreal classification. A total of 257 patients with IBD were included in the study, of which 126 had UC and 131 had CD. Results: Of the 257 patients with IBD, 134 (52.2%) were male and 123 (47.8%) were female. The mean age of patients with CD and UC were 28.74 (range 15–67) and 38.79 (range 15–75) years, respectively. There was a significant difference between all age groups (*p* < 0.005), with more patients aged over 40 years being diagnosed with UC. UC was most commonly classified as left-sided UC (E2) (60; 47.6%), while the most common location of CD was the ileocolon (L3) (76; 58%). Non-stricturing and non-penetrating CD was the most common behavior (B1) (70; 53.8%). Approximately one-third of the patients with Crohn’s disease developed perianal disease (70; 27.2%), with fistulizing Crohn’s being the most prevalent manifestation (40; 30.5%), followed by abscess formation (10; 7.6%) and fissures (9; 6.8%). The most common extraintestinal manifestation was arthropathy (15; 5.8%). Significant *p*-values were detected for UC and CD (*p* = 0.036). Conclusions: This study provides crucial insights into the demographics, patterns, and manifestations of IBD in Saudi Arabia. The findings highlight the need for improved diagnosis, management, and treatment strategies for IBD in this population. The establishment of a multi-center database will help to facilitate future research and improve patient care in Saudi Arabia.

## 1. Introduction

Crohn’s disease (CD) and ulcerative colitis (UC), collectively referred to as inflammatory bowel diseases (IBD), are chronic immune-mediated gastrointestinal tract disorders [1]. The etiology of IBD remains unknown, however, the disease is thought to be caused by a complex interaction of genetic and environmental factors, including dietary components and tobacco use, resulting in the inappropriate activation of the mucosal immune system driven by a loss of tolerance to gut commensal bacteria [2,3].

The prevalence of IBD is currently the highest in Europe (UC, 505 per 100,000 persons; CD, 322 per 100,000 persons) and North America (UC, 249 per 100,000 persons; CD, 319 per 100,000 persons) [4]. Its global prevalence is rising, with rapid increases in incidence rates being observed as more countries adopt Westernized lifestyles [5,6]. In 2010, the EpiCom/Epi-IBD study was launched to explore the epidemiology and management of IBD in Eastern and Western European countries. This study included 1515 IBD patients and found an incidence rate of 15 cases per 100,000 person-years [7]. In Spain, the overall cumulative incidence of IBD, CD, and UC during the first year of follow-up was 16.2, 7.4, and 8.1 cases per 100,000 person-years, respectively, across 108 hospitals that served over 22 million inhabitants [8].

Data regarding the exact prevalence and incidence of IBD in Saudi Arabia are limited, as there are few published studies in this regard. However, a retrospective analysis of patients within two decades (1983–2022) at King Saud University Hospital in Riyadh, published by Al-Ghamdi et al., demonstrated an annual incidence of CD of 0.94 per 100,000 population per year, while another study published by El-Mouzan et al. showed an annual incidence of 0.5 per 100,000 population per year, with a prevalence of 5 per 100,000 population per year in the pediatric age group [9]. Both studies were limited in incidence and prevalence owing to the lack of adequate sample sizes of 77 and 50, respectively, as well as being limited to a single region. The first study published in 1982 indicated that inflammatory bowel disease was rare or nonexistent in Saudi Arabia [1]. In the following years, multiple studies have suggested an increase in the incidence of IBD in Saudi Arabia [10,11,12,13,14,15].

We aimed to establish a comprehensive understanding of IBD trends in the Qassim region by compiling data from multiple centers. This data would include information about the clinical diagnostic features of CD and UC, baseline characteristics, natural history, location, and behavior of IBD in the region. The study aimed to compare this data with international statistics to identify patterns and trends in IBD occurrence and management.

## 2. Materials and Methods

This retrospective study included patients diagnosed with inflammatory bowel disease at King Fahad Specialist Hospital-Burydah (KFSH-B), King Saud Hospital Onaizah (KSU), and Buraidah Central Hospital (BCH) from 22 April 2014 to 30 November 2018. We collected data on patients’ characteristics, disease location, behavior, age at diagnosis (according to the Montreal classification), anti-TNF use, surgical complications related to IBD, and extraintestinal manifestations.

### 2.1. Inclusion and Exclusion Criteria

**Figure 1:**
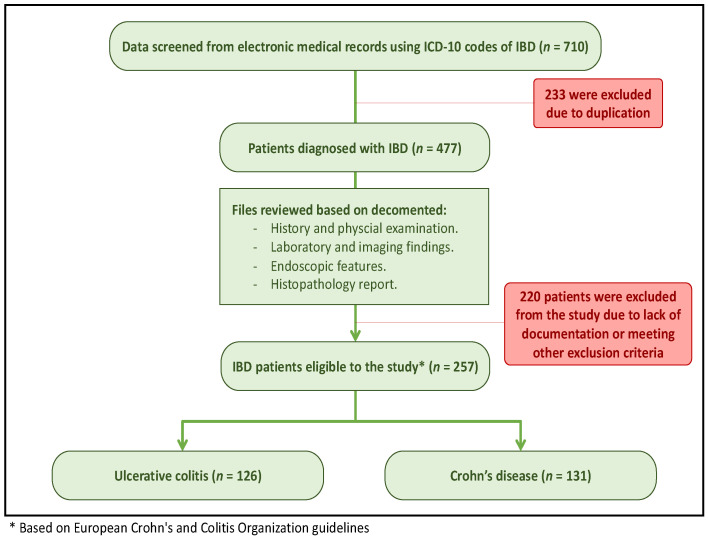
Study design depicting the selection of IBD patients from electronic medical records using ICD-10 codes as well as the final number of patients included in the analysis.

The study included adult Saudi patients with documented endoscopic or radiological (CT enterography) features along with histopathological findings of either CD or UC. All samples underwent an assessment for the typical histopathological features used in the diagnosis of UC and CD, which include crypt architecture distortion, granulomas, identification of histologic changes in areas of normal endoscopy, and a search for dysplasia. In addition, a virology workup was performed during any admission. Patients who did not meet these criteria were excluded from the study based on specific exclusion criteria, which included patients younger than 14 years of age, those with inconclusive or insufficient diagnostic data for IBD, and non-Saudi patients. These exclusion criteria helped to ensure that the study’s findings were representative of the clinical characteristics of CD and UC in the target patient population (Figure 1).

### 2.2. Data Collection Methods

Data were collected from the medical records of patients with IBD into a checklist fulfilling the following variables: age, diagnosis, time from presentation to diagnosis, sex, body mass index (BMI) at diagnosis, and family history of IBD. We also collected data on comorbidities, baseline laboratory investigations at the time of diagnosis (hemoglobin (Hb), mean corpuscular volume (MCV), mean corpuscular hemoglobin concentration (MCHC), erythrocyte sedimentation rate (ESR), C-reactive protein (CRP), liver function tests (LFTs)), and Montreal classification of the extent of UC and CD. Furthermore, CT enterography and MR enterography (MRE) techniques were utilized, when available, as part of the methodology for evaluating the characteristics of Crohn’s disease and ulcerative colitis, in order to further characterize the tissue.

### 2.3. Study Sample

The study included a total of 257 patients, with 190 patients from KSFH-B, 37 patients from KSU, and 30 patients from BCH. These patients were selected based on specific inclusion and exclusion criteria, and their files and data were reviewed by two residents in the Department of Gastroenterology. Duplicate files were identified and removed from the study. The files and data of the selected patients underwent independent review by two residents from the gastroenterology department to ensure the accuracy and reliability of the data.

### 2.4. Quality Assessment

The research team implemented quality control measures to minimize bias in the study’s design, conduct, and analysis. Frequent meetings were held to critically appraise each step of the study and identify potential sources of bias. Two independent reviewers evaluated each component of the study, including the data collection, analysis, and interpretation of results. Any discrepancies or disagreements were resolved through open discussions and consensus-building among the research team. These measures ensured the validity and reliability of the study’s findings, providing confidence in its conclusions and increasing the credibility and generalizability of the results.

### 2.5. Data Analysis

The data collected for the study were entered into Microsoft Excel and then transferred to SPSS version 23.0 (IBM Corp., Armonk, NY, USA) for analysis. Numerical variables were analyzed by calculating the mean, median, and standard deviation (SD), while categorical variables were presented as numbers and percentages. Categorical variables were compared using the Chi-square (*χ*^2^) test and Fisher’s exact test, while quantitative variables were analyzed using the appropriate test. Appropriate statistical methods were applied to compare variables such as age, extraintestinal manifestations, anti-tumor necrosis factor (anti-TNF) use, and surgical rates between Crohn’s disease (CD) and ulcerative colitis (UC). These methods helped determine the significance of the observed differences between the two groups and contributed to the overall analysis of the study’s findings.

## 3. Results

**Figure 2:**
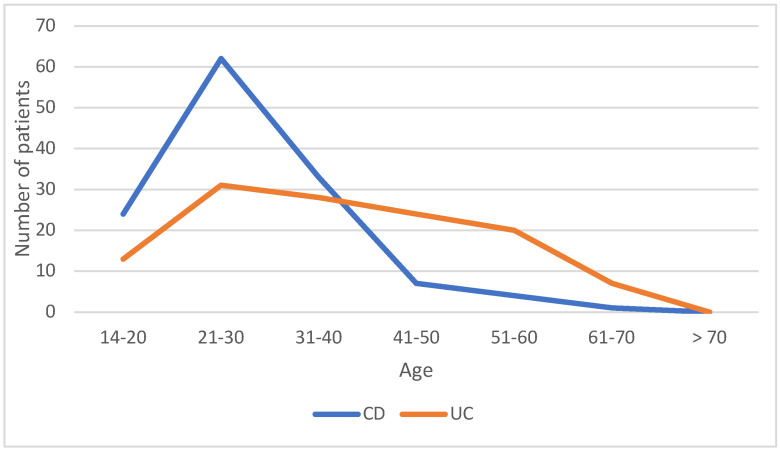
Distribution of Crohn’s disease and ulcerative colitis in the different age groups.

A total of 257 patients with IBD were included in the study from 22 April 2014 to 30 November 2018. Almost half of the patients (*n* = 126; 49%) had UC and the other half (*n* = 131; 51%) had CD (Table 1). The mean age of patients with CD was 28.74 years and patients with UC was 38.79 years. The age range for patients with CD was from 15 to 67 years and the range for patients with UC was from 15 to 75 years. The most prevalent age groups presenting with UC were the 17–40 years (*n* = 68, 53.9%) age group, >40 years (*n* = 54, 42.8%), and the <17 years (*n* = 4, 3.2%) age groups (Figure 2). On the other hand, the most common age group for CD was the 17–40 years (*n* = 117; 89.3%) age group, followed by >40 and <17 years age groups (*n* = 11; 8.4%; *n* = 3; 2.3%,), respectively, as shown in Table 1. There was a significant difference between all age groups (*p* < 0.005), with more patients aged >40 years being diagnosed with UC. A total of 134 (52.2%) patients were male and 123 (47.8%) were female. Regarding disease distribution, according to gender in UC patients, 66 (52.4%) patients were male and 60 (47.6%) patients were female. In the CD group, 68 (52%) patients were male and 63 (48%) patients were female. No significant difference between the sexes was detected (*p* = 0.939).

Approximately one-third of the patients developed perianal disease (*n* = 70; 27.2%) (*p* < 0.005). Among the CD group, fistulizing Crohn’s was the most prevalent among perianal disease (*n* = 40; 30.5%), followed by abscess formation (*n* = 10; 7.6%) and fissures (*n* = 9; 6.8%). In the UC group, a low incidence of perianal disease, including fistula and fissures, was detected (*n* = 11; 8.7%). Significant *p*-values were detected across all variables, as presented in Table 1, except for abscess formation (*p* = 0.418).

**Figure 3:**
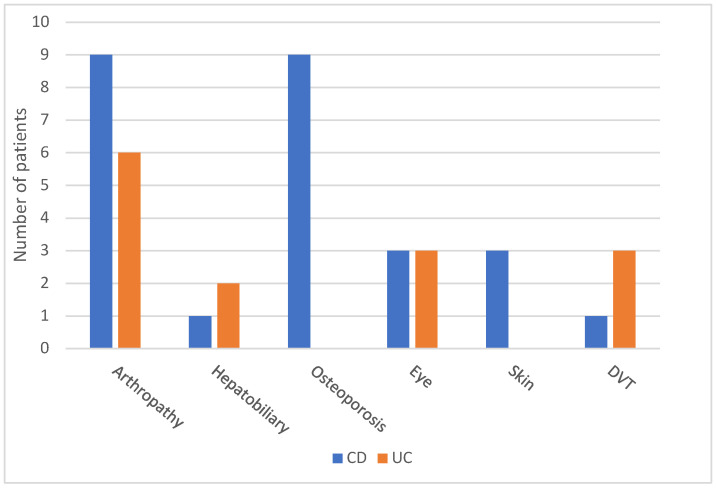
Different extraintestinal manifestations of both Crohn’s disease and ulcerative colitis.

Approximately 18% of patients with IBD developed extraintestinal manifestations (*n* = 45; 17.5%). The specifications of the extraintestinal manifestations are shown in Table 1 and Figure 3. The most common extraintestinal manifestation was arthropathy (*n* = 15; 5.8%). Among the 15 patients with IBD, arthropathy was more common in the CD group than in the UC group. Six patients developed peripheral arthritis (*n* = 6; 4.7%), but only two of these patients had CD (*n* = 2; 1.5%). Significant *p*-values were detected for UC and CD (*p* = 0.036). More cases of sacroiliitis were noted in patients with CD (*n* = 7; 5.3%) than in those with UC. Regarding primary sclerosing cholangitis (*n* = 3; 1.1%), most of the patients with these conditions had UC (*n* = 5; 3.9% and *n* = 2; 1.5%, respectively) (see Table 1).

Thromboembolic events (*n* = 4; 1.5%) were highest in the UC group (*n* = 3; 2.3%) than in the CD group (*n* = 1; 0.8%). Overall, the incidence of osteoporosis was highest in patients with CD (*n* = 9; 6.9%), and no patient with UC had osteoporosis (*p* = 0.024; Table 1 and Figure 3).

Regarding eye diseases in patients with IBD (*n* = 6; 2.3%) who developed eye manifestations, a total of three patients (*n* = 3; 2.3%) in both groups were noted with insignificant differences. Skin manifestation in patients with IBD (*n* = 3; 1.1%) who developed symptoms was highest in patients with CD (*n* = 3; 2.3%), with a significant difference between the two groups of IBD.

A total of 100 (38.9%) patients with IBD were taking either budesonide or prednisone, which are commonly used as steroids in our hospitals. Most patients with CD received steroids (*n* = 85; 44.2%), while less than half of patients with UC received steroids (Table 1). A total of 42 (33.3%) patients with IBD were on anti-TNF (*n* = 121; 47%). Most patients with CD received anti-TNF (*n* = 86; 65.6%), while only a few patients (*n* = 35; 27.8%) with UC were on anti-TNF (Table 1). A significant difference was noted between categorical variables (*p* < 0.005). A significant difference was seen in patients who underwent abdominal surgery for IBD, especially CD (*n*= 43; 32.8%), compared with those for UC (*n* = 3; 2.3%; *p* < 0.005).

Regarding the classification of CD based on the Montreal classification, for the age groups at diagnosis, most cases were in the 17–40 years age group (A2) (*n* = 107; 81.7%), followed by the <16 years age groups (A1) (*n* = 17; 13%), and lastly the >40 years (A3) (*n* = 7; 5.3%) age group Table 1.

Regarding CD location, the most common was the ileocolonic region ((L3) (*n* = 76; 58%; and ilial (L1) (*n* = 34; 26%)), followed by the colonic region (L2) (*n* = 19; 14.5%), and isolated upper disease (*n* = 2; 1.5%). For CD behavior, the non-stricturing and non-penetrating (B1) type was the most common (*n* = 70; 53.8%), followed by the stricturing (B2) type (*n* = 31; 23.8%), and lastly is the penetrating disease (B3) (*n* = 29; 22.3%).

Classification of UC based on the Montreal classification, left-sided UC (E2) (*n* = 60; 47.6%) was the most common type of UC according to the Montreal classification, followed by ulcerative proctitis (E1) (*n* = 34; 27%), and the least common was extensive UC (pancolitis) (E3) (*n* = 32; 25.4%) (Table 1).

No significant association between abscesses, fissures, fistulas, and CD was observed (Table 2). Cross tabulation between CD and abscess formation showed that there was 80% more abscess formation in the ileocolon (L3) (*n* = 8; 80%) (Table 2). Most of the patients with fissures or fistulas in the CD group had these lesions in the ileocolonic area ((L3) (*n* = 4; 44%) and (*n* = 23; 57.5%)), respectively (Table 2).

A significant difference was noted in CD behavior and abscess formation, as well as fistula creation, with *p*-values of 0.012 and 0.004, respectively. The most common behavior was the penetrating behavior (B3) in patients with CD, presenting with more abscesses (*n* = 6; 60%), fistulas (*n* = 16; 40%), and fissures (*n* = 4; 44%) (Table 3).

Approximately 50% of patients presenting with stricturing (B2) (*n* = 16; 51.6%) and penetrating (B3) (*n* = 12; 41.4%) Crohn’s disease had previous surgery with a significant *p*-value of 0.007 (Table 4).

## 4. Discussion

This was a retrospective study of the clinical data of 257 patients with IBD. We showed that there is a limited predominance of CD over UC, which differs from previously reported data from Saudi Arabia that showed a significant predominance of CD [16,17]. This also differs from reports on North American populations, which indicate a predominance of UC over CD [18,19].

Before 1982, inflammatory bowel disease (IBD) was considered rare in the Middle East, especially in Saudi Arabia [17]. However, a 6-year study conducted in Kuwait in 1984 challenged this view by reporting 91 cases of ulcerative colitis (UC) and 17 cases of Crohn’s disease (CD) in the region [17,20]. Subsequent studies conducted in the 1990s and 2000s in countries such as Saudi Arabia, Kuwait, and Oman have confirmed an increasing incidence of IBD in the region [17,20,21]. For instance, a case series study conducted in Egypt from 1995 to 2009 found 135 UC patients and 22 CD patients among the 24,156 patients referred to a gastroenterology center [21]. Recent reports have also confirmed a significant rise in the incidence of IBD in Arab countries. For example, a study in a tertiary center in Riyadh, Saudi Arabia found that the incidence of CD increased from 0.32 to 1.66 per 100,000 persons per year between 1983–2004, with a total mean annual incidence of 0.94 per 100,000 persons per year over 20 years [9]. According to Mosli et al.’s recent meta-analysis, the incidence of inflammatory bowel disease (IBD) in the Arab world was estimated to be approximately 2.33 and 1.46 per 100,000 persons per year for ulcerative colitis (UC) and Crohn’s disease (CD), respectively [22]. Based on the population of the Qassim region, which is approximately 1,423,935 [23], the estimated number of individuals with ulcerative colitis (UC) and Crohn’s disease (CD) would be approximately 33 and 21, respectively, per year, according to Mosli et al.’s recent meta-analysis. Similar numbers were found over 4 years in our study with a slightly high CD over UC, 31.5 and 32.75, respectively, per year.

Regarding sex differences in IBD, the data in our study showed that there was a male predominance in both CD and UC, which is consistent with previously reported data in Saudi Arabia [16]. However, this difference was not significant (*p* = 0.939). These data are different from those in North America and Europe, which show a female predominance in CD and a male predominance in UC [24,25].

The reported median age at presentation of CD in Saudi Arabia varies among centers. In one study it was 23.8 years [17], while another study reported that the median patient age at presentation was 27.15 years and the mean age at diagnosis in North America is approximately 30 years [26]. In this study, the median age of patients with UC was higher (38.79 years) than the age reported in previous studies from our country and North America [16,17,25], while the median age of patients with CD was 28.74 years.

Our data show that in 17.5% of patients with IBD that developed extraintestinal manifestations, arthropathy involvement was the most common, followed by skin manifestations and eye manifestations, similar data were reported in our country and North America [16,27,28,29]. A significant difference was noted in the prevalence of arthritis and osteoporosis between patients with CD and those with UC across the age groups, as shown in Table 1. Similar results were observed by Adam et al., as they reported more cases of primary sclerosing cholangitis and skin involvement, likely due to the nature of the tertiary care center as a referral center for more complex cases.

Thromboembolism occurred at a rate of 1.5% (*n* = 4) in IBD, while a rate of 0.8% (*n* = 1) was observed in patients with CD, and 2.3% (*n* = 4) in patients with UC. In our study, we observed a lower prevalence of thromboembolism than what was reported in the literature (Alberene, 2% for CD; Adam et al., 3.8% for IBD; and similar reported rates of venous thromboembolism were reported in North America 1.22%) [28,29,30].

According to the Montreal classification, most of our patients had ileocolonic involvement (*n* = 76, 58%), with only 1.5% (*n* = 2) of cases being confined to the colon. In patients with CD, the non-stricturing and non-penetrating (B1) behavior was the most common (*n* = 70, 53.4%), and this is similar to the report by Aljebreen et al. However, our data is different from those of some Western countries in terms of the behavior of CD according to the course of the disease [27,28,31].

Data regarding UC involvement in this study differ from data published in our country and in North America, as our findings show that the main type of UC is the left-sided type (*n* = 60, 47.6%), followed by proctitis (*n* = 34, 27%), but pancolitis being the least, while it is the most common type in the literature [15,19]. There were no significant differences in disease behavior or location with respect to age at diagnosis between the UC and CD groups.

Overall, 27.2% of our patients had a perianal disease and 16.7% had fistulae, which is similar to the data by Aljebreen et al. and is also consistent with the figures from most local and international published studies (20–30% and 15–20%, respectively) [28,32,33,34,35]. We found a significant association between perianal disease, especially abscess formation, and fistula formation in relation to CD behavior (*p* = 0.012 and *p* = 0.004, respectively), with an overall incidence of 38.1% (Table 1, Table 2 and Table 3). Among patients with a perianal disease in the form of abscesses, approximately 60% had the penetrating type of Crohn’s disease (B3), and around 40% had fistula formation, according to our data.

This study showed that there was a significant increase in the use of anti-TNF for both UC and CD compared to the previously reported data, which could be attributed to advances in pharmacotherapy and changes in recent guidelines [17]. Furthermore, 38.9% of our patients were on steroids as either steroid-dependent or independent cases, which is lower than what was reported in the study by Aljebreen et al. [28].

In this study, the related rate of surgery was significant for both UC and CD (2.3% and 32%, respectively), which is similar to the data reported in our country and in North America, but the surgery rates for UC were lower [17,28,36,37].

The study had several limitations that may affect the generalizability and interpretability of its findings. Firstly, the sample size was relatively small, with only 257 patients with IBD included in the analysis. This limited sample size may not be representative of the broader population of patients with IBD and may also affect the statistical power of the study. Consequently, the findings of the study should be interpreted with caution, and further research with a larger sample size may be necessary to confirm the study’s results. Secondly, the study was retrospective, which may have led to incomplete or inaccurate data collection. Retrospective studies rely on data collected from medical records, which may be incomplete, inconsistent, or missing information. This limitation may have affected the validity and reliability of the data used in the study, potentially leading to biased or inaccurate results.

Finally, the study did not investigate the impact of treatment on disease outcomes. Although the study aimed to examine the demographic and clinical factors associated with IBD, the lack of information on treatment efficacy may limit the practical implications of the study’s findings. Future studies may need to explore the impact of different treatments on IBD outcomes to provide a more comprehensive understanding of the disease and its management.

## 5. Conclusions

IBD is commonly seen in this region, with CD and UC predominantly affecting younger men. Our study identified differences in disease characteristics, including location and behavior based on the Montreal classification, when compared to international data. Furthermore, we observed a notable rise in the use of anti-TNF medications for both CD and UC.

## Figures and Tables

**Table 1:** Patients’ baseline characteristics and key manifestations.

Variables	Overall *n* = 257 (100%)	UC *n* = 126 (49%)	CD *n* = 131 (51%)	$\chi^{2}$	*p*-Value
Demographics					
Mean age (SD), years	33.6	38.79 (14.9)	28.74 (9.16)		
<17	7 (2.7%)	4 (3.2%)	3 (2.3%)	41.48	<0.005
17–40	185 (71.9%)	68 (53.9%)	117 (89.3%)		
>40	65 (25.2%)	54 (42.8%)	11 (8.4%)		
Gender					
Male	134 (52.1%)	66 (52.4%)	68 (52%)	0.005	0.939
Female	123 (47.8%)	60 (47.6%)	63 (48%)		
Extent of UC					
E1, *n* (%)	---	34 (27%)	---		
E2, *n* (%)	---	60 (47.6%)	---		
E3, *n* (%)	---	32 (25.4%)	---		
Age at CD diagnosis					
A1, *n* (%)	---	---	17 (13%)		
A2, *n* (%)	---	---	107 (81.6%)		
A3, *n* (%)	---	---	7 (5.3%)		
CD location					
L1, *n* (%)	---	---	34 (26%)		
L2, *n* (%)	---	---	19 (14.5%)		
L3, *n* (%)	---	---	76 (58%)		
L4, *n* (%)	---	---	2 (1.5%)		
CD behavior					
B1, *n* (%)	---	---	70 (53.4%)		
B2, *n* (%)	---	---	32 (24.4%)		
B3, *n* (%)	---	---	29 (22.2%)		
Perianal disease					
All, *n* (%)	70 (27.2%)	11 (8.7%)	59 (45%)	42.72	<0.005
Fistula, *n* (%)	43 (16.7%)	3 (2.3%)	40 (30.5%)	6.4259	0.011
Fissure, *n* (%)	14 (5.4%)	5 (3.9%)	9 (6.8%)	5.285	0.021
Abscess, *n* (%)	13 (5%)	3 (2.3%)	10 (7.6%)	0.653	0.418
Extraintestinal manifestation					
All, *n* (%)	45 (17.5%)	19 (17.4%)	26 (23.6%)	1.010	0.314
Eye, *n* (%)	6 (2.3%)	3 (2.3%)	3 (2.3%)	0.200	0.653
Skin, *n* (%)	3 (1.1%)	0 (0%)	3 (2.3%)	0.546	0.459
Arthropathy					
Peripheral arthritis, *n* (%)	8 (3.1%)	6 (4.7%)	2 (1.5%)	4.352	0.036
Sacroiliitis, *n* (%)	7 (2.7%)	0 (0%)	7 (5.3%)	3.265	0.070
Hepatobiliary					
PSC, *n* (%)	3 (1.1%)	2 (1.5%)	1 (0.8%)	0.828	0.362
Thromboembolic, *n* (%)	4 (1.5%)	3 (2.3%)	1 (0.8%)	1.998	0.154
Osteoporosis, *n* (%)	9 (3.5%)	0 (0%)	9 (6.9%)	5.040	0.024
Management					
Steroids, *n* (%)	100 (38.9%)	42 (33.3%)	58 (44.2%)	3.234	0.072
Anti-TNF, *n* (%)	121 (47%)	35 (27.7%)	86 (65.6%)	36.971	<0.005
Related surgeries, *n* (%)	46 (17.8)	3 (2.3%)	43 (32.8%)	40.506	<0.005

**Table 2:** Association of Crohn’s disease location in relation to perianal manifestation.

Abscess in Crohn’s Disease in Relation to the Location												
			L1		L2		L3		L4			
			N	%	N	%	N	%	N	%	N	
Abscess	No		33	27.3%	18	14.9%	68	56.2%	2	1.7%	121	0.516
	Yes		1	10.0%	1	10.0%	8	80.0%	0	0.0%	10	
Total			34	26.0%	19	14.5%	76	58.0%	2	1.5%	131	
		Fistula in Crohn’s Disease in Relation to the Location									Total	*p*
		L1		L2		L3		L4				
		N	%	N	%	N	%	N	%		N	
Fistula	No	22	24.2%	14	15.4%	53	58.2%	2	2.2%		91	0.705
	Yes	12	30.0%	5	12.5%	23	57.5%	0	0.0%		40	
Total		34	26.0%	19	14.5%	76	58.0%	2	1.5%		131	
		Fissure in Crohn’s Disease in Relation to the Location									Total	*p*
		L1		L2		L3		L4				
		N	%	N	%	N	%	N	%		N	
Fissure	No	32	26.2%	16	13.1%	72	59.0%	2	1.6%		122	0.412
	Yes	2	22.2%	3	33.3%	4	44.4%	0	0.0%		9	
Total		34	26.0%	19	14.5%	76	58.0%	2	1.5%		131	

**Table 3:** Association of Crohn’s disease behavior in relation to perianal manifestation.

		Fissure in Crohn’s Disease in Relation to Its Behavior						Total	*p*
		B1		B2		B3			
		N	%	N	%	N	%	N	
Fissure	No	68	56.2%	28	23.1%	25	20.7%	121	0.117
	Yes	2	22.2%	3	33.3%	4	44.4%	9	
Total		70	53.8%	31	23.8%	29	22.3%	130	
		Abscess in Crohn’s Disease in Relation to Its Behavior						Total	*p*
		B1		B2		B3			
		N	%	N	%	N	%	N	
Abscess	No	67	55.8%	30	25%	23	19.2%	120	0.012
	Yes	3	30%	1	10%	6	60%	10	
Total		70	53.8%	31	23.8%	29	22.3%	130	
		Fistula in Crohn’s Disease in Relation to Its Behavior						Total	*p*
		B1		B2		B3			
		N	%	N	%	N	%	N	
Fistula	No	55	61.1%	22	24.4%	13	14.4%	90	0.004
	Yes	15	37.5%	9	22.5%	16	40.0%	40	
Total		70	53.8%	31	23.8%	29	22.3%	130	

**Table 4:** Association of Crohn’s disease behavior in relation to previous surgeries.

		Previous Surgery				Total	*p*
		No		Yes			
		N	%	N	%	N	
Crohn’s Disease Behavior	B1	55	78.6%	15	21.4%	70	0.007
	B2	15	48.4%	16	51.6%	31	
	B3	17	58.6%	12	41.4%	29	
Total		87	66.9%	43	33.1%	130	

## Data Availability

Data are available upon requests submitted to the authors.
